# A repeat cross-sectional study examining the equitable impact of nutritional standards for school lunches in England in 2008 on the diets of 4-7y olds across the socio-economic spectrum

**DOI:** 10.1186/s12966-014-0128-6

**Published:** 2014-10-24

**Authors:** Suzanne Spence, John NS Matthews, Martin White, Ashley J Adamson

**Affiliations:** Institute of Health and Society, Newcastle University, Newcastle upon Tyne, UK; Human Nutrition Research Centre, Newcastle University, Newcastle upon Tyne, UK; School of Mathematics and Statistics, Newcastle University, Newcastle upon Tyne, UK; Fuse, UKCRC Centre for Translational Research in Public Health, Newcastle upon Tyne, UK

**Keywords:** School lunch, Packed lunch, Children, Inequalities, Nutrition

## Abstract

**Background:**

The 2008 nutritional standards for primary school lunch in England improved nutritional content. The impact on socio-economic inequalities is unknown. We examine the impact of the nutritional standards on children’s nutrient intake at lunchtime and in total diet by level of deprivation.

**Methods:**

We conducted cross-sectional studies in 12 English primary schools before and after legislation. Dietary intake was recorded for 4-7y olds using a validated, prospective four-day food diary. Socio-economic status was estimated using the Index of Multiple Deprivation; three groups of approximately equal sizes were created. Linear, mixed-effect models explored the effect of year, lunch type (school or home-packed lunch), level of deprivation and the interaction(s) between these factors on children’s diets.

**Results:**

368 and 624 children participated in 2003–4 and 2008–9 respectively. At lunchtime, between 2003–4 and 2008–9, the increase in non-starch polysaccharide (NSP) intake was larger in the least compared to the most deprived group (difference in mean change 0.8 mg; 95% CI 0.4, 1.3). There were similar differences in mean changes for iron (0.3 mg; 0.2, 0.4) and zinc (0.3 mg; 0.1, 0.5). In total diet, differential effects were observed for NSP, iron and zinc; we found no evidence these changes were associated with lunch type. Lunch type was associated with changes in per cent energy from non-milk-extrinsic sugars (NMES) and vitamin C. Per cent energy from NMES was lower and vitamin C intake higher in school lunches in 2008–9 compared with 2003–4. The corresponding differences in home-packed lunches were not as marked and there were subtle but statistically significant effects of the level of deprivation.

**Conclusions:**

By 2008–9, NMES at lunchtime and in total diet was lower for children consuming a school lunch; this change was equitable across the deprivation groups. Vitamin C intake increased more for children in the most deprived group, narrowing the socio-economic inequality. A range of significant differential effects of the nutritional standards were observed and important socio-economic inequalities in dietary intake remain. Additional interventions to promote equitable nutrition in children are needed to support legislative measures and maximise their impact.

## Background

Dietary intake has an important influence on child health [1,2] but there remain important socio-economic inequalities [3]. Identifying solutions has proved challenging [4-6] because children’s diets are influenced by many individual, social and environmental factors [1,7].

The school environment has long been considered important in the promotion of child health nationally and internationally [8,9], and there is increasing evidence for the effectiveness of school interventions to tackle obesity [10-12]. Schools are important environments for dietary interventions, due to the time children spend in school, exposure to school food [12] and their potential to influence food choice and behaviours [13-15] across the socio-economic spectrum [16].

Primary schools in England have been required to comply with legislation detailing specific food- and nutrient-based standards for school food since September 2008 [17,18]. This was in response to a number of factors; for example, national surveys of school lunch in primary and secondary schools [19,20] and a media broadcast in April 2005 *“Jamie’s School Dinners”* [21] highlighted the poor nutritional content of school lunch. The introduction of these standards to school lunches aimed to improve children’s dietary intake at lunchtime. Several studies have reported improvements in children’s mean nutrient intake from a school lunch associated with the introduction of the food and nutrient-based standards [22,23]. In a recent study we examined the impact of this legislation on children’s mean intake at lunchtime and in total dietary intake. Our key findings showed a widening difference in mean macro- and micronutrient intakes between a school and home-packed lunch, with the average school lunch providing a ‘healthier’ option. Improvements were also found for children consuming a school lunch in their mean total dietary intake [24]. However, it is not known if the changes to school lunch impact equitably across the socio-economic spectrum, for example, does improving food provision at school lunch inadvertently increase the difference in children’s mean nutrient intake due to individual food choice? As the standards focus only on school lunch, what is the impact of home-packed lunch on nutrient intake across the socio-economic spectrum? With the recent UK Government announcement that all children aged 4–7 years in England will be entitled to a free school lunch from September 2014 [25], understanding further the impact of school lunch on children’s diets across the socio-economic spectrum is important.

The primary aim of this paper is to examine the impact of the 2008 food and nutrient-based standards on socio-economic inequalities in food consumed at lunchtime and in total diet in children aged 4-7years. A secondary aim is to examine the change in school lunch take-up across deprivation groups.

## Methods

Details of the methods have been previously reported [24,26]; a brief summary is provided below.

### Setting and participants

Cross-sectional studies were undertaken in primary schools in Newcastle, North East England over two academic years: 2003–4, n = 16 (before) and 2008–9, n = 13 (after implementation of the legislation). The 2003–4 data were collected as part of a previous study [27] and used as baseline. The analysis presented includes data collected from 12 schools that participated in both 2003–4 and 2008–9. This was a key aspect for this study; to recruit the same schools for which we had dietary data pre-implementation of the policy to enable us to compare nutrient intake pre and post-implementation. Schools were originally selected in 2003–4 using the free school meal index [28] as a proxy measure for the level of deprivation in the school population to seek a balance across the socio-economic spectrum. The free school meal index indicates the percentage of children in a school eligible for free school meals. The schools that participated were selected to cover a range of deprivation areas in Newcastle; Newcastle consists of 26 wards with varying levels of deprivation (Index of Multiple Deprivation (IMD) range: 7.56 to 75.57), the schools that participated were from 9 wards with a range in IMD: 7.56 to 73.92. The same schools were invited to participate in 2008–9; only after consent by Head teachers were schools included. After parental consent individual level IMD was determined from postcodes and used in the analysis. All children in reception, year 1 and 2 were eligible to participate. Parents provided informed, written consent prior to children participating and ethical approval was granted by Newcastle University Ethics Committee (reference 000011/2007).

### Data

#### Dietary

We used identical dietary data collection methods in 2003–4 and 2008–9. Using a previously validated prospective four-day food diary (the *Food Assessment in Schools Tool* (*FAST*)) [24,27], we recorded children’s dietary intake over three consecutive week days and one weekend day (Wednesday to Saturday inclusive). Parents received written instructions on how to complete the diary at home. At each school a team of trained observers and the study nutritionist recorded dietary intake. Foods were categorised into ‘school’, ‘home-packed lunch’, and ‘food consumed at home’. Dietary coding for nutritional composition was based on McCance and Widdowson’s Integrated Composition of Food Dataset [29]. The specific macro- and micronutrients examined in this paper relevant to the nutrient-based standards are: energy, per cent energy from fat, saturated fat and non-milk intrinsic sugars (NMES), and absolute amounts of non-starch polysaccharide (NSP), iron, zinc and vitamin C. Children’s mean nutrient intakes were compared to the nutrient-based standards [30] at lunchtime and to dietary reference values [31] for total diet.

School lunches were coded using school lunch recipes, made available by relevant primary school catering services.

#### Socio-economic

Socio-economic status (SES) was estimated using the English Index of Multiple Deprivation (IMD) 2007, matched to full (7 digit) postcodes at the Lower Layer Super Output area level for individual children’s home address [32]. IMD is a composite measure of deprivation including seven domains; income, employment, health and disability, education, skills and training, barriers to housing and services, crime and living environment [32]. This enables areas to be ranked by relative deprivation [32]. The IMD scores were then categorised into three groups of approximately equal size for the analyses: group 1 included children living in the 20% least deprived areas, group 2 children living in the 60% mid-deprived areas, and group 3 included children living in the 20% most deprived areas.

### Statistical analysis

The analyses examined the change in school lunch take up and children’s mean macro- and micronutrient intakes at lunchtime and in total diet.

Logistic regression was used to examine the change in school lunch take up by year and level of deprivation. The analysis examined the effect of year (before and after legislation), a child’s lunch type (school or home-packed lunch), level of deprivation (least, mid and most deprived groups), as factors and the interaction(s) between these factors. We used a linear mixed effect model, with year, lunch type, level of deprivation and gender taken as fixed effects. Potential correlation between responses within the same school or child were accommodated by fitting random effects for each. The models were fitted using ‘lme’ in R (version 2.14.0). Data for vitamin C were log-transformed because of skewness and geometric means are reported.

### Variables

Main outcome measures were change in mean daily intakes of macro- and micronutrients in school and home-packed lunch, and total diet by level of deprivation. Macro- and micronutrients reported in this paper are: energy (kcals), per cent energy from fat, saturated fat and non-milk extrinsic sugars, non-starch polysaccharides (g), iron (mg), zinc (mg) and vitamin C (mg). Predictors were year, lunch type and level of deprivation.

## Results

### Participants and school lunch take up in 2003–4 and 2008–9

**Figure 1:**
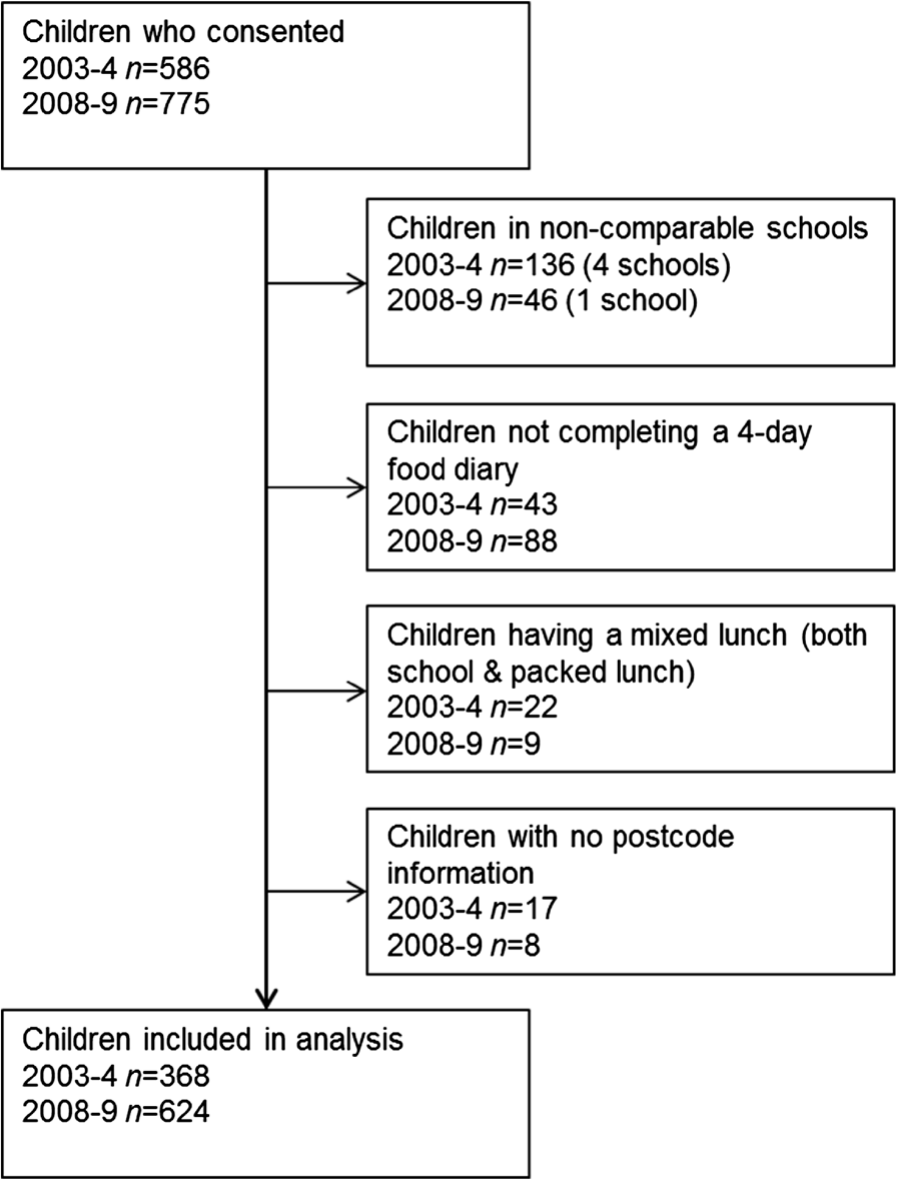
**Flowchart for number of children consenting, reasons for exclusion and final number included in analysis.**

The analyses included 368 children in 2003–4 (63% of those consenting) and 624 (81% of those consenting) in 2008–9; reasons for exclusion are shown in Figure 1. There were similar numbers of boys and girls participating in 2003–4 (male *n* = 181 (49%); female *n* = 187 (51%)) and 2008–9 (male *n* = 317 (51%); female *n* = 307 (49%)), mean age was 5.8y in 2003–4 and 6.1y in 2008–9. We found no statistically significant difference in the level of deprivation for children included in the analysis in 2003–4 and 2008–9 (mean IMD 27.0 and 26.1 respectively, p = 0.50) (Table 1).

**Table 1:** **Study sample characteristics**

	**2003-4**		**2008-9**	
	***(n*** **= 368)**		***(n*** **= 624)**	
	**(Male = 181; Female = 187)**		**(Male = 317; Female = 307)**	
	**Mean**	**SD**	**Mean**	**SD**
Age	5.8	0.7	6.1	0.9
Index of Multiple Deprivation	27.0	20	26.1	21

Between 2003–4 and 2008–9, there was a decrease in the percentage of children consuming a school lunch across all deprivation groups (p = 0.005; Table 2); the odds ratio (OR) for consuming a school lunch in 2008–9 compared with 2003–4 OR was 0.68 (95% CI 0.52 to 0.88). Children in the most deprived group were more likely to have a school lunch compared with those in the mid and least deprived groups (p <0.001, OR 1.41, 1.23 to 1.62). There was no evidence of any interaction between year by level of deprivation (p = 0.38), indicating no change in the relationship between level of deprivation and school lunch take-up over time.

**Table 2:** **Number (percentage) of children consuming a school lunch by year and level of deprivation**

	**2003-4**	**2008-9**
**Level of deprivation:**	***n*** **(%)**	***n*** **(%)**
Least deprived	43 (54)	81 (42)
Mid deprived	90 (54)	105 (50)
Most deprived	89 (74)	132 (60)
All children	222 (60)	318 (51)

### Children’s mean nutrient intake

#### Lunchtime

##### Level of deprivation

Children in the least deprived group had a higher mean energy intake (520 kcals) at lunchtime compared with those in the mid and most deprived groups (mid = 487 kcals, least deprived = 492 kcals; p = 0.002), regardless of year or lunch type.

##### Year by level of deprivation interaction

**Figure 2:**
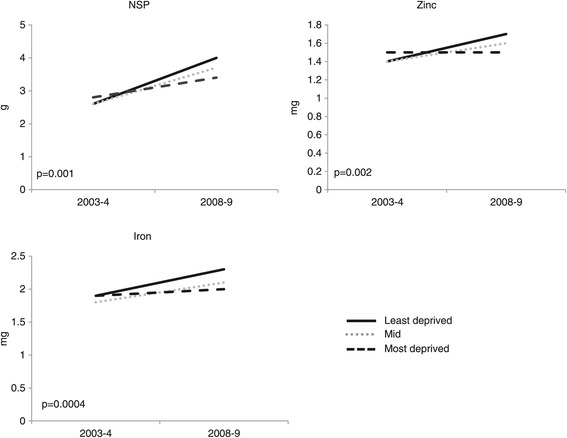
**The effect of year by level of deprivation on children’s mean nutrient consumption of NSP, iron and zinc at lunchtime (adjusted for lunch type and gender).**

We found no evidence of a year by level of deprivation interaction in relation to children’s mean intake of per cent energy from fat (p = 0.7), saturated fat (p = 0.7), non-milk-extrinsic sugars (NMES) (p = 0.4) or vitamin C intake (p = 0.6). In 2003–4, there was little difference in children’s mean NSP, iron or zinc intake between deprivation groups (Figure 2 and Table 3). Between 2003–4 and 2008–9, mean NSP intake increased in all deprivation groups; the mean change was greatest in the least deprived group (year by level of deprivation interaction, p = 0.001; Figure 2). Between 2003–4 and 2008–9, mean iron and zinc intake increased in the least and mid-deprived groups, but there was little change in the most deprived group (year by level of deprivation interaction, p = 0.0004 and p = 0.002 respectively; Figure 2 and Table 3). These changes were not associated with lunch type.

**Table 3:** **Lunchtime: the effect of year by level of deprivation on children’s mean nutrient intake compared with nutrient-based standards** [30]

			**2003-4**	**2008-9**	**[2008–9] – [2003–4]**	
**Nutrient**	**Standard**	**Level of deprivation**	Mean*		**(Mean change) 95%** **confidence interval**	**p-value for interaction** ^**†**^ **(year by level of deprivation)**
NSP^±^ (g)	Min 4.2	Least	2.6	4.0	(1.4) 1.1,1.8	
		Mid	2.6	3.7	( 1.1) 0.9,1.4	
		Most	2.8	3.4	(0.6) 0.3,0.9	0.001
Iron (mg)	Min 3	Least	1.9	2.3	(0.4) 0.3,0.6	
		Mid	1.8	2.1	(0.3) 0.2,0.4	
		Most	1.9	2.0	(0.1) -0.1,0.2	0.0004
Zinc (mg)	Min 2.5	Least	1.4	1.7	(0.3) 0.2,0.4	
		Mid	1.3	1.6	(0.3) 0.1,0.4	
		Most	1.5	1.5	(0.0) -0.1,0.1	0.002
Vitamin C (mg)*	Min 10.5	Least	16.5	33.4	(0.49) 0.40,0.61	
		Mid	14.2	32.6	(0.44) 0.37,0.51	
		Most	14.3	31.9	(0.45) 0.37,0.54	0.64

*Arithmetic means are reported, except for vitamin C (highly skewed) where geometric means and ratios are given.

^†^p-value for interaction derived from a linear mixed effects model with random term for schools.

^±^NSP (non-starch polysaccharide).

##### Lunch by level of deprivation

We found no evidence of any lunch by level of deprivation interactions on the nutrients examined.

Although children in the least deprived group had a higher mean NSP, iron and zinc intake, mean intakes remained below the nutrient-based standards for school lunch of 4.2 g, 3 mg and 2.5 mg respectively [30], regardless of whether they consumed a school or home-packed lunch.

#### Total diet

##### Year by level of deprivation interaction

**Figure 3:**
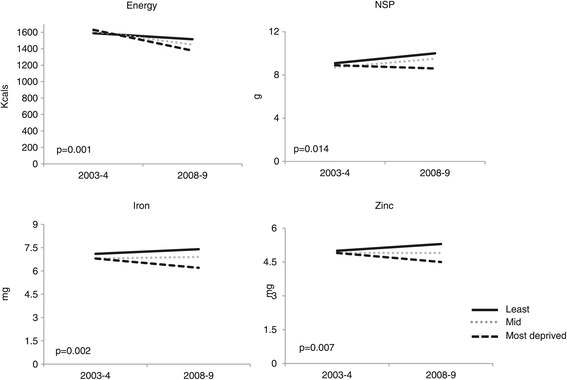
**The effect of year by level of deprivation on children’s mean nutrient consumption of energy, NSP, iron and zinc in total diet (adjusted for lunch type and gender).**

Between 2003–4 and 2008–9, there was a decrease in mean energy intake in total diet in all deprivation groups (year by level of deprivation interaction, p = 0.001; Figure 3); this decrease was smallest in the least deprived group (−73 kcals) and largest in the most deprived (−253 kcals).

Children’s mean per cent energy from fat and saturated fat both improved (decreased) from 2003–4 and 2008–9, but there was no evidence of a year by level of deprivation interaction (p = 0.4 and p = 0.06 respectively) (Table 4). In 2003–4 and 2008–9, children’s mean intake of per cent energy from fat was below the recommended guideline level of 35%, but above the recommended level of 11% for saturated fat [31].

**Table 4:** **Total diet: the effect of year by level of deprivation on children’s mean nutrient intake compared with DRV/RNI’s** [31]

			**2003-4**	**2008-9**	**[2008–9] – [2003–4]**	
**Nutrient**	DRV/RNI*	**Level of deprivation**	**Mean**		**(Mean change) 95%** **confidence interval**	**p-value for interaction** ^**†**^ **(year by level of deprivation)**
Energy (kcals)	^±^	Least	1589	1516	(−73) -143,-2.2	
		Mid	1612	1450	(−162) -216,-107	
		Most	1630	1377	(−253) -315,-121	0.001
% E fat^**ǂ**^	35	Least	33.3	31.5	-(1.8) -2.8,-0.8	
		Mid	34.5	31.5	(−3.0) -3.4,-1.9	
		Most	34.1	31.9	(−2.2) -3.0,-1.4	0.4
% E saturated fat^**ǂ**^	11	Least	14.4	13.8	(−0.6) -1.2,0.0	
		Mid	14.8	13.4	(−1.4) -1.8,-0.9	
		Most	14.1	13.4	(−0.7) -1.2,-0.2	0.06
NSP^**≠**^ (g)	-	Least	9.1	10.0	(0.9) 0.3,1.6	
		Mid	8.7	9.5	(0.8) 0.3,1.3	
		Most	8.9	8.6	(−0.3) -0.9,0.4	0.014
Iron (mg)	6.1	Least	7.1	7.4	(0.3) -0.7,0.1	
		Mid	6.8	6.9	(0.1) -0.4,0.2	
		Most	6.8	6.2	(−0.6) -1.0,-0.3	0.002
Zinc (mg)	6.5	Least	5.0	5.3	(0.3) -0.1,0.5	
		Mid	4.9	4.9	(0.0) -0.2,0.2	
		Most	4.9	4.5	(−0.4) -0.6,-0.1	0.007

*Dietary reference value/Reference nutrient intake [31].

^†^p-value for interaction derived from a linear mixed effects model with random term for schools.

^±^Boy (1715 kcals), Girl (1545 kcals).

^**ǂ**^% E fat/saturated fat (per cent energy derived from fat/saturated fat).

^**≠**^NSP (non-starch polysaccharides).

In 2003–4, there was little difference in children’s mean NSP, iron and zinc intake between deprivation groups. Between 2003–4 and 2008–9, there was an increase in mean NSP intake in the least and mid-deprived groups, but a decrease in the most deprived group (year by level of deprivation interaction, p = 0.014; Figure 3). Between 2003–4 and 2008–9, there was little change in children’s mean iron and zinc intake in the least and mid-deprived groups, but a fall in intake for children in the most deprived group (year by level of deprivation interactions: p = 0.002 and 0.007 respectively) (Figure 3). These changes were not associated with lunch type. Across all levels of deprivation, children’s mean iron intake met the reference nutrient intake of 6.1 mg/day; mean zinc intake was below the recommended 6.5 mg/day [31].

##### Level of deprivation, year and lunch type interaction

**Figure 4:**
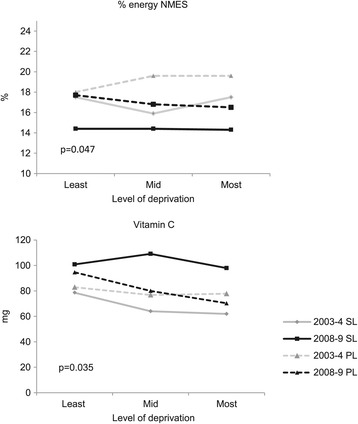
**The effect of level of deprivation, year and lunch type on children’s mean per cent energy NMES and vitamin C intake in total diet (adjusted for gender).**

In total diet a significant interaction between level of deprivation, year and lunch type was found for two nutrients: per cent energy from NMES (p = 0.047) and vitamin C (p = 0.035) (Figure 4). In 2003–4, children from across the deprivation groups who ate a school lunch had a lower per cent energy (%E) from NMES compared with children who ate a home-packed lunch (Figure 4 and Table 5). The difference between a school and home-packed lunch in the least deprived group was 0.5%E and in the most deprived group 2.1%E. Between 2003–4 and 2008–9, per cent energy NMES intake from school lunch fell and children who ate a school lunch continued to have a lower intake. For children who ate a home-packed lunch, mean intake remained similar between 2003–4 and 2008–9 in the least deprived group (mean change −0.3%) but fell in the most deprived group (−3.1%) (difference in mean change −2.8%; 95% CI −5.5 to −0.1). This led to an improvement in mean percent energy from NMES in all deprivation groups for children consuming a school lunch, but a disparity for children consuming a home-packed lunch with higher levels in the least deprived group. Across all groups, children’s mean per cent energy NMES remained above the dietary reference value of 11% in their total diet [31].

**Table 5:** **Total diet: the effect of level of deprivation, year and lunch type on per cent energy NMES and mean vitamin C intake**

		**School lunch**			**Packed lunch**			
		**2003-4**	**2008-9**	**[2008–9] – [2003–4]**	**2003-4**	**2008-9**	**[2008–9] – [2003–4]**	
**Nutrient**	**Level of deprivation**	Mean*		**(Mean change) 95%** **Confidence Interval**	**Mean**		**(Mean change) 95%** **Confidence Interval**	**p-value for interaction** ^**†**^
% energy NMES	Least	17.5	14.4	(−3.1) -4.9,-1.3	18.0	17.7	(−0.3) -2.1,1.5	
	Mid	15.9	14.4	(−1.5) -2.9,-0.1	19.6	16.8	(−2.8) -4.2,-1.4	
	Most	17.5	14.3	(−3.2) -4.5,-1.8	19.6	16.5	(−3.1) -5.1,-1.1	0.047
Vitamin C (mg)*	Least	78.6	100.8	(0.8) 0.7,0.9	82.9	94.6	(0.9) 0.7,1.1	
	Mid	64.0	109.1	(0.6) 0.5,0.7	76.8	80.0	(1.0) 0.8,1.1	
	Most	61.9	97.9	(0.6) 0.5,0.7	77.8	70.3	(1.1) 0.9,1.3	0.035

*Arithmetic means are reported, except for vitamin C (highly skewed) where geometric means and ratios are reported.

^†^p-value for 3-way interaction derived from a linear mixed effects model with random term for schools.

In 2003–4, children who ate a school lunch had a lower mean vitamin C intake in all deprivation groups compared with children who ate a home-packed lunch (Figure 4 and Table 5). The difference between children having a school and home-packed lunch in the least deprived group was −4.3 mg and in the most deprived group −15.9 mg. In 2008–9, children who ate a school lunch had a higher mean vitamin C intake, which was similar in the least and most deprived groups; the increase was smaller in the least deprived group (22.2 mg) compared with the most deprived group (36 mg; Figure 4). For children who ate a home-packed lunch, mean intake increased in the least deprived group (11.7 mg) but fell in the most deprived group (−7.5 mg), leading to a wider difference between lunch type in the least deprived group (Figure 4). Across the deprivation groups, children’s mean vitamin C intake met the reference nutrient intake of 30 mg/day in 2003–4 and 2008–9 [31].

## Discussion

### Summary of key findings

In 2008–9, following legislation to introduce nutritional standards for primary school lunches in England, school lunch take-up decreased across all deprivation groups. Between 2003–4 and 2008–9, our findings show a widening difference by level of deprivation in mean NSP, iron and zinc intakes at lunchtime and in total diet, but we found no evidence this was influenced by lunch type. In total diet, year, lunch type and level of deprivation were found to influence children’s mean per cent energy from NMES and vitamin C, and there was a widening difference by lunch type. For children consuming a school lunch per cent energy from NMES reduced to similar levels for all the deprivation groups thereby narrowing inequalities, whereas for children consuming a home-packed lunch, the decrease was less marked in the least deprived group. For children consuming a school lunch children’s vitamin C intake was now similar, leading to a narrowing of socio-economic inequalities; in contrast, for children consuming a home-packed lunch there was a widening of socio-economic inequalities, with children from the least deprived families now having a substantially higher intake.

### Strengths and limitations

We used identical dietary data collection methods in 2003–4 and 2008–9 to avoid introducing measurement bias [20]. Key strengths of this dietary data collection method are that it was previously validated, is easy for parents to use and all food consumed was observed [27]. This limited the problems associated with dietary self-report methods in this age-group.

Previous studies [21,22] have only collected data post-legislation with no baseline against which to assess the impact of change at lunchtime or in total diet. This is the first study to use a natural experimental [33], repeat cross-sectional design to evaluate the 2008 legislation to improve the nutritional content of school food in England, and to analyse differential impact according to socio-economic status. A limitation of such a design is attributing causality. In addition, this study cannot account for other secular changes that may be associated with changes in diet such as national campaigns or the economic climate. However, we have reported change in intake from school lunch which can be attributed to a change in school food policy. As previously reported, national implementation of the food- and nutrient-based standards imposed time constraints that prevented the use of a stronger study design with prospective follow-up of individual children [23]. This study was also limited to 12 primary schools in Newcastle in the North East of England, so, findings may not be generalisable.

For children consuming a school lunch we had no information on free or paid school lunch at an individual level; this could have been advantageous for a more detailed analysis examining the impact of lunch type (free and paid school lunch) on children’s total diet.

A limitation of using IMD is that it does not measure individual socio-economic status, and is therefore subject potentially to the ecological fallacy [34] resulting in misclassification bias [34].

### Relationship to previous work

Socio-economic differences in diet are well established; children from more deprived families have been found to consume more energy dense [35-38] and less nutrient-dense foods [39]. Factors such as availability, accessibility [40], parental education and income [37], and cost of foods have been identified as contributing factors [41].

In 2010, a study found a statistically significant difference in children aged 3-17y mean total dietary intake of per cent energy NMES across socio-economic groups; children in the least deprived group consumed less [42]. In our study, between 2003–4 and 2008–9, we found a statistically significant difference between deprivation groups in children’s mean total dietary intake of per cent energy NMES. But, in contrast, between 2003–4 and 2008–9, we found children consuming a home-packed lunch in the least deprived group had a higher mean intake of NMES compared with those in the most deprived group; for children consuming a school lunch there was a similar intake across the deprivation groups. A key difference between these studies was that we examined the impact by lunch type.

Findings from a cross-sectional study using data from the low income diet and nutrition survey collected between 2003 and 2005 did not find any significant differences in energy or nutrient intake between those having a school or home-packed lunch over the whole day [43]. In contrast, we found some evidence that, following the introduction of nutritional standards, between 2003–4 and 2008–9 a child’s lunch type had an impact on mean total nutrient intake across levels of deprivation (e.g. per cent energy NMES and vitamin C). However, we were not able to differentiate between children who ate free or paid school lunches, nor limit the analysis to only those children in the most deprived groups, which may explain some of the differences in our findings compared with those previously published [43].

A study in Texas using a pre- (2001–2) and post-policy (2005–6) evaluation in middle schools found reductions in children’s mean energy density intake (2.08 kcal/g to 2.17; p <0.0001) in school lunches associated with policy changes [44]. Changes included restrictions to portion sizes of certain foods and drinks, fat content, and frequency of provision [14,44]. In addition, they examined the effect of socio-economic status across schools and observed the greatest changes in schools from the higher and mid-socio-economic areas [44]. In our analysis we did not examine energy density, but we found there was no statistically significant impact of school level variation, and therefore we assessed the impact of deprivation at an individual rather than school level.

### What this study adds

There is evidence to suggest that legislation to improve the nutritional content of school lunches has been effective overall [22-24,45,46]. However, this is the first study to examine whether the changes following the 2008 legislation introducing nutritional standards for school lunches in English primary schools had a similar impact on children’s diets across levels of deprivation. Our findings for lunchtime suggest that the least deprived children are consuming more nutrient-dense foods from both school lunch and home-packed lunch compared with the most deprived children. Despite this, even for children in the least deprived group, mean NSP, iron and zinc intakes remained below the nutrient-based standards of 4.2 g, 3 mg and 2.5 mg respectively [30]. This highlights children’s dietary intake from either a school or home-packed lunch needs to be addressed across the socio-economic spectrum, but most urgently in children from the most deprived families. We found evidence of widening inequalities in children’s mean NSP, iron and zinc intake in total diet; however, there was no evidence lunch type influenced this. Nevertheless, within the limitations of this study there is some evidence that lunch type influences socio-economic inequalities in children’s total diet. Legislative changes affecting nutritional content of school lunches were associated with an improvement in per cent energy NMES intake across the deprivation groups; and mean vitamin C intake improved more for the most deprived children, leading to a narrowing of socio-economic inequality.

### Implications for policy, practice and further research

Although legislation introducing nutritional standards for school lunches has the potential to improve children’s diets, consideration must be given to the possibility that population-based interventions may be differentially effective across socio-economic groups and may have other unintended consequences [47,48].

The findings of this study show where we found evidence of an improvement in children’s total dietary intake associated with regulation of the nutritional content of school lunches, for example per cent energy NMES; this benefitted children equally. Although vitamin C intake improved more for the most deprived children, this policy change benefitted children across the social spectrum, and there was a levelling in inequalities. However, we also found that, despite the introduction of legislation to improve the nutritional content of school lunches, there was a widening in inequalities in children’s mean NSP, iron and zinc intakes at lunchtime and in total diet. These findings suggest that to achieve its full potential, regulation of nutritional standards for school lunches may need to be supplemented by additional behavioural interventions [49] to improve children’s food choice at school lunch, particularly for those in the most deprived groups. Guidance aimed at parents and children’s food choices when preparing and consuming home-packed lunches is also required. The finding that children in the least deprived group consuming a home-packed lunch post-legislation have a higher per cent energy from NMES may be due to a higher consumption of products such as smoothies and fruit juices, perceived as ‘*healthy’*; this reinforces the need for parental awareness of nutritional content of products [42].

An unintended outcome of implementing the food and nutrient-based standards may be the subsequent decrease in school lunch take-up. While this decrease may in part be attributable to cost and increasing pressures on family budgets, this study found a decrease in school lunch take-up across levels of deprivation. Free school meals are to be introduced for all children in England aged 4–7 years from September 2014 [25], which is expected to increase take-up. However, it is not known whether free school meals will be taken up equally by all, or whether this intervention may potentially widen or narrow inequalities in children’s diets. Further detailed and robust prospective evaluation is needed. Future policy changes to school food in England, such as the equity impacts of the universal free school lunch, need to consider evaluation outcomes prior to implementation. A whole school approach which goes beyond change in provision and encourages children’s food choice may offer a potential solution to inequalities in food choice [50]. The findings from this study suggest that interventions to supplement the regulation of school food, which considers social and economic factors beyond the school environment, are needed to address the complexity of inequalities in children’s total dietary intake [51,52].

## Abbreviations

FAST: Food Assessment in Schools Tool
IMD: Index of Multiple Deprivation
SES: Socio-economic status
OR: Odds ratio
CI: Confidence interval
NSP: Non-starch polysaccharides
NMES: Non-milk extrinsic sugars
g: Grams
mg: Milligrams
kcals: Kilocalories
%E: Per cent energy
